# Clear Cell Renal Cell Carcinoma is linked to Epithelial‐to‐Mesenchymal Transition and to Fibrosis

**DOI:** 10.14814/phy2.13305

**Published:** 2017-06-08

**Authors:** Lea Landolt, Øystein Eikrem, Philipp Strauss, Andreas Scherer, David H. Lovett, Christian Beisland, Kenneth Finne, Tarig Osman, Mohammad M. Ibrahim, Gro Gausdal, Lavina Ahmed, James B. Lorens, Jean Paul Thiery, Tuan Zea Tan, Miroslav Sekulic, Hans‐Peter Marti

**Affiliations:** ^1^ Department of Clinical MedicineUniversity of BergenBergenNorway; ^2^ Department of MedicineHaukeland University HospitalBergenNorway; ^3^ SpheromicsKontiolahtiFinland; ^4^ Institute for Molecular Medicine Finland (FIMM)University of HelsinkiHelsinkiFinland; ^5^ Department of MedicineSan Francisco VAMCUniversity of California San FranciscoSan FranciscoCalifornia; ^6^ Department of UrologyHaukeland University HospitalBergenNorway; ^7^ BerGenBio ASBergenNorway; ^8^ Department of BiomedicineCenter for Cancer BiomarkersUniversity of BergenBergenNorway; ^9^ INSERM UMR 1186Integrative Tumor Immunology and Genetic OncologyGustave RoussyEPHEFac. de médecine‐Univ. Paris‐SudUniversité Paris‐SaclayVillejuifFrance; ^10^ Science Institute of SingaporeNational University of SingaporeSingaporeSingapore

**Keywords:** Clear cell renal cell carcinoma, epithelial‐to‐mesenchymal transition, fibrosis

## Abstract

Clear cell renal cell carcinoma (ccRCC) represents the most common type of kidney cancer with high mortality in its advanced stages. Our study aim was to explore the correlation between tumor epithelial‐to‐mesenchymal transition (EMT) and patient survival. Renal biopsies of tumorous and adjacent nontumorous tissue were taken with a 16 g needle from our patients (*n* = 26) undergoing partial or radical nephrectomy due to ccRCC. RNA sequencing libraries were generated using Illumina TruSeq^®^ Access library preparation protocol and TruSeq Small RNA library preparation kit. Next generation sequencing (NGS) was performed on Illumina HiSeq2500. Comparative analysis of matched sample pairs was done using the Bioconductor Limma/voom R‐package. Liquid chromatography‐tandem mass spectrometry and immunohistochemistry were applied to measure and visualize protein abundance. We detected an increased generic EMT transcript score in ccRCC. Gene expression analysis showed augmented abundance of *AXL* and *MMP14*, as well as down‐regulated expression of *KL* (klotho). Moreover, microRNA analyses demonstrated a positive expression correlation of miR‐34a and its targets *MMP14* and *AXL*. Survival analysis based on a subset of genes from our list EMT‐related genes in a publicly available dataset showed that the EMT genes correlated with ccRCC patient survival. Several of these genes also play a known role in fibrosis. Accordingly, recently published classifiers of solid organ fibrosis correctly identified EMT‐affected tumor samples and were correlated with patient survival. EMT in ccRCC linked to fibrosis is associated with worse survival and may represent a target for novel therapeutic interventions.

## Introduction

Renal cell carcinoma, also named renal cell adenocarcinoma, comprises over 80% of primary renal neoplasms and is among the ten most frequent forms of cancer in both men and women (Escudier et al. 2016). Clear cell renal cell carcinoma (ccRCC) represents the most common (75–85%) form of renal cell carcinomas and is one of the most lethal genitourinary cancers. Up to a quarter of patients display distal metastases or advanced regional disease at the time of presentation and diagnosis.

Morbidity and mortality of advanced ccRCC is high. Patients with metastatic ccRCC have a 5‐year survival of between 0 and 32% depending upon their risk stage at time of diagnosis (Escudier et al. 2016). Therefore, the identification of new mechanisms, biomarkers and related treatment targets is of great clinical importance for the management of ccRCC patients.

Our study focuses on the role of epithelial‐to‐mesenchymal transition (EMT) as one of the initial steps toward the development of fibrosis, and its potential role in ccRCC. EMT was first described in the 1980s and is physiologically involved in embryogenesis and in pathological states, such as development of solid organ fibrosis. EMT is also associated with tumor invasiveness and distal metastasis (Gjerdrum et al. 2010; Thiery and Lim 2013; Lovisa et al. 2015; Piva et al. 2016). EMT defines a process, where epithelial cells lose their polarity and barrier integrity and develop a mesenchymal phenotype, which includes acquired motility. The acquisition of a mesenchymal phenotype in part results from the loss of intercellular junctions and from the reorganization of the actin cytoskeleton to promote migratory behavior (Thiery and Sleeman 2006; Kidd et al. 2014). Moreover, EMT renders cancer cells immune‐evasive, drug‐resistant, and contributes to the metastatic cascade (Gjerdrum et al. 2010; Fleuren et al. 2014; Reichl et al. 2015; Zhou et al. 2016b). Thus, activation of EMT is a key process that promotes local invasion, distal metastasis and drug resistance (Gjerdrum et al. 2010). The identification of the signaling pathways leading to activation of EMT programs in cancer should provide new insights into cell plasticity and therapeutic interventions to optimize health care delivery. Accordingly, EMT has been shown to play a key role in the progression of both experimental and human ccRCC (Yu et al. 2015; Piva et al. 2016; Zhou et al. 2016a).

Different molecules and pathways are associated with the control of EMT, including transforming growth factor *β* (TGF*β*), fibroblast growth factor (FGF), and klotho (KL) (Thiery and Sleeman 2006; Doi et al. 2011; Grande et al. 2015). Known EMT triggers are cytokines such as TGF*β* and FGF. TGF*β* and EGF lead to the activation of transcriptional factors including SNAIL1 and SNAIL2, TWIST, ZEB1 and ZEB2 which induce gene expression patterns favoring EMT development (Lamouille et al. 2014; Grande et al. 2015; Lovisa et al. 2015). As a consequence, down‐regulation of E‐cadherin (CDH1), an epithelial marker involved in intercellular connections, and up‐regulation of vimentin (VIM), a mesenchymal marker, are typically observed in EMT. However, carcinoma cells can adopt multiple intermediate, possibly metastable stages (Nieto et al. 2016).

The transcription factors SNAIL1, ZEB1, and ZEB2 induce the expression of matrix metalloproteinases (MMPs). MMPs are crucial mediators of cancer EMT as they influence tumor behavior, especially invasiveness, by proteolysis of extracellular matrix (ECM) (Kalluri and Neilson 2003; Jorda et al. 2005; Thiery et al. 2009; Mahimkar et al. 2011; Lamouille et al. 2014). The principal MMPs involved in cancer are MMP2, MMP9 and most notably MMP14 (Seiki et al. 2003; Mahimkar et al. 2011; Lamouille et al. 2014). Increased expression of MMP2 and MMP9 is associated with poor prognosis in ccRCC (Kallakury et al. 2001; Chen et al. 2014; Mikami et al. 2016). Expression of MMP14, also known as MT1MMP, correlates with the extent of renal epithelial tumor EMT and invasive capacity (Mahimkar et al. 2011). Mutation or hypermethylation‐induced inactivation of the tumor suppressor gene Von‐Hippel‐Lindau (VHL) increases activity of the mitogen‐activated protein kinase (MEK) protein. Sustained activation of the MEK1 module leads to the higher expression of MMP14 and is related to the degree of EMT. In accordance, the activity of the MEK1/MMP14 signaling module is highly correlated with tumor nuclear grade and invasiveness of ccRCC (Mahimkar et al. 2011).

The AXL receptor tyrosine kinase, with its main ligand growth arrest‐specific 6 (GAS6), is emerging as another important promotor and regulator of EMT (Gjerdrum et al. 2010). High expression of AXL and its pathway can be detected in various cancers including acute myeloid leukemia, prostate, breast, lung, and skin cancers (Axelrod and Pienta 2014). AXL, along with MERTK and TYRO3, is a member of the TAM receptor family (Korshunov 2012). Binding of GAS6 leads to the oligomerization of AXL with tyrosine phosphorylation and activation of a down‐stream signaling cascade (Jorda et al. 2005). AXL‐mediated EMT is known to be important for ccRCC progression (Yu et al. 2015; Zhou et al. 2016b).

Both AXL and MMP14 mRNA's are targeted by miR‐34a, which represents an important microRNA in cancer development (Jia et al. 2014; Li et al. 2015). MiR‐34a represents a mediator of p53‐dependent tumor inhibition, and its low expression correlates with worse prognosis in several cancers (Fritz et al. 2015).

The aim of this study, which includes mRNA and to a more limited extent miRNA sequencing and proteomics, is to describe in detail the dysregulation of EMT and its related genes in a cohort of ccRCC patients and to determine if the results have prognostic value in other patient cohorts.

## Methods

### Patients and renal tissues

This study includes renal tissue from 26 patients with ccRCC who attended Haukeland University Hospital in Bergen Norway and underwent partial or radical (full) nephrectomy from November 2013 until September 2015, as published previously (Eikrem et al. 2016a,2016b). Two additional patients were excluded because of a mixture of carcinoma and normal tissue in the respective biopsies. Renal core biopsies of tumorous and adjacent noncancerous normal tissue, as judged by histopathology, were taken with a 16 g needle perioperatively. Core biopsies of tumorous and adjacent non‐tumorous tissues were formalin‐fixed and paraffin‐embedded (FFPE), as described (Eikrem et al. 2016a).

Patients (10 females and 16 males) had a mean age of 57.4 ± 12 years (58.2 ± 11 years for females and 56.9 ± 12.9 years for males). All patients had tumor‐negative lymph nodes and no metastases. Detailed patient characteristics are summarized in Table 1.

**Table 1:** Patient characteristics

Patient ID	Age (year)	Gender	BMI (kg/m^2^)	Nephrectomy type	eGFR (mL/min/1.73 m^2^)	TNM‐stage	Fuhrmann grade (1–3)	Cancer stage (1–4)	Leibovich score (0–11)	Sample usage
9	70	Male	24	Partial	>60	pT1AcN0cM0	2	1	0	R
10	69	Male	34	Partial	>60	pT3AcN0cM0	2	2	4	R
11	37	Male	27	Partial	>60	pT1AcN0cM0	2	1	0	R
13	63	Male	24	Full	40	pT3AcN0cM0	4	2	8	R
15	68	Male	28	Partial	>60	pT1AcN0cM0	2	1	0	R
18	78	Male	27	Full	47	pT3AcN0cM0	2	2	4	R, P
19	71	Female	22	Full	>60	pT2aN0cM0	1	2	4	R
21	53	Female	25	Full	55	pT1 BcN0cM0	2	1	2	*R, P*
22	49	Male	25	Partial	>60	pT1 BcN0cM0	2	1	3	*R, P*
27	46	Male	31	Full	>60	pT2BcN0cM0	3	2	5	*R, P*
29	54	Female	29	Partial	>60	pT1AcN0cM0	2	1	0	*R, P*
31	67	Male	25	Partial	>60	pT1AcN0cM0	1	1	0	*R, P*
32	36	Male	23	Partial	>60	pT1AcN0cM0	3	1	1	R
33	48	Male	28	Partial	>60	pT1AcN0cM0	1	1	0	*R, P*
39	71	Male	25	Full	59	pT3AcN0cM0	4	3	8	R, M, P
44	74	Female	23	Full	>60	pT3AcN0cM0	2	3	4	R, M, P
46	53	Female	24	Partial	>60	pT1AcN0cM0	1	1	0	R, M, P
50	72	Female	19	Full	>60	pT1 BcN0cM0	2	1	3	R, M
53	46	Female	44	Full	>60	pT2AcN0cM0	2	2	3	R, M, P
55	44	Female	23	Full	>60	pT3AcN0cM0	3	3	5	R, M
57	63	Female	28	Full	>60	pT1AcN0cM0	2	1	0	R, M
59	52	Female	29	Partial	>60	pT1AcN0cM0	2	1	0	R, M
63a	55	Male	28	Partial	>60	pT1AcN0cM0	3	1	1	R, M
63b	44	Male	20	Partial	>60	pT1AcN0cM0	2	1	0	R, M
64	52	Male	26	Full	>60	pT1 BcN0cM0	3	1	4	R, M
65	57	Male	24	Full	>60	pT2AcN0cM0	3	2		R, M

Characteristic patient (*n* = 26) features at the time of surgery. eGFR was calculated with the MDRD formula. Full nephrectomy is equivalent to radical nephrectomy. The “Cancer Stage” was determined based on the European Association of Urology (EAU) Guidelines on renal cell carcinoma: 2014 update (Gjerdrum et al. 2010). Leibovich score: 1 = low risk, 2 = intermediate risk, 3 = high risk (Leibovich et al. 2003). Sample usage: “R”, mRNA sequencing; “M”, microRNA (miR) sequencing; “P” , proteomics.

The regional Ethics Committee of Western Norway approved our studies (REC West no. 78/05). All participants provided written consent as requested by our Ethics Committee.

### RNA extraction and quality assessment for next generation sequencing

Total RNA was extracted from FFPE stored kidney core biopsies using the miRNeasy FFPE kit (Qiagen) as reported (Eikrem et al. 2016a; Landolt et al. 2016). Quality and quantity of extracted RNA were assessed using the Agilent RNA 6000 Nano Kit on a 2100 Bioanalyzer instrument (Agilent Technologies, Santa Clara, CA).

We calculated the DV200 value (the percentage of RNA fragments longer than 200 nucleotides) as a measure to estimate the quality of RNA necessary for sequencing (Huang et al. 2015; Eikrem et al. 2016a; Landolt et al. 2016). The mean DV_200_ values (95% CI) of the samples of 26 patients (with each tumorous and non‐tumorous FFPE samples) were 65.7% (58.9–72.4%); a minimum level of 30% is required for sequencing (Huang et al. 2015; Eikrem et al. 2016a).

### RNA library preparation and sequencing

RNA sequencing libraries were prepared using the TruSeq RNA Access library kit (Illumina, Inc., San Diego, CA, USA) for mRNA sequencing (*n* =  all 26 patients) or the TruSeq Small RNA Library Preparation Kits (Illumina Inc, San Diego, CA) for miRNA sequencing (subgroup of *n* = 12 patients, as shown in Table 1) according to the manufacturer`s protocol. RNA sequencing was performed on a HiSeq2500 instrument (Illumina, San Diego, CA) according to the manufacturer's protocol and as described previously (Eikrem et al. 2016a,2016b).

Sequencing data are available via Gene Expression Omnibus, https://www.ncbi.nlm.nih.gov/geo/; GSE76207 and GSE82122.

### Bioinformatics of RNA sequencing

Assembly of reads and alignment of the contigs to the Human Genome Assembly GRCh38 were guided by Tophat and Bowtie. For the mRNA expression data of 52 samples from 26 patients, an empirical expression filter was applied, which left genes with at least 3 counts per million (cpm) in at least 19 samples.

A list of 483 EMT genes was compiled from three publically available sources (http://dbemt.bioinfo-minzhao.org; (Chen et al. 2014; Groger et al. 2012)), and supplemented by the addition of genes involved in EMT based on our own study interest, as described below.

For the microRNA expression data of 22 samples from 11 patients, an empirical expression filter was applied, which left microRNA species with at least 3 cpm in at least nine samples.

Comparative analysis was done using the voom/Limma R‐package. Differential gene expression with a moderated paired *t*‐test was defined as Benjamini‐Hochberg adjusted *P* ≤ 0.05, and an absolute fold change of ≥2.

Survival analyses using mRNA or microRNA were performed using two tools, (http://bioinformatica.mty.itesm.mx:8080/Biomatec/SurvivaX.jsp), and SurvMicro (http://bioinformatica.mty.itesm.mx:8080/Biomatec/Survmicro.jsp), respectively. For the mRNA analysis we used 468 samples from the TCGA ccRCC dataset; for the microRNA analysis we employed 217 HiSeq samples from TCGA. Further data analysis and visualization was performed with JMP Pro 11 (www.sas.com), and Graphpad version 6 and 7 (www.graphpad.com).

Classifier analysis was performed as recently published (Rodder et al. 2009, 2011). The classifier consisted of a panel of metzincins and related genes (MARGS), and the algorithm linear discriminant analysis. The analysis was performed in JMP Pro 11 (www.sas.com). The ccRCC dataset was treated as the test set, as the classifiers had been trained already in other datasets (Rodder et al. 2009, 2011).

### Transcriptomic EMT score

A generic EMT score was computed for each of the RNAseq samples using a method described previously (Tan et al. 2014). Briefly, using an EMT signature derived from bladder, breast, colorectal, lung, gastric, and ovarian cancers, a two‐sample Kolmogorov‐Smirnov test was performed to assess the extent of EMT enrichment. A high enrichment score of the EMT signature indicate a sample to be more mesenchymal‐like, whereas a low enrichment score indicates a sample to be more epithelial‐like.

### Proteomics

This investigation was performed in a subgroup of patients (*n* = 11) from our total subject cohort (*n* = 26), as indicated in Table 1. Protein and peptide extraction: After deparaffinization with xylene, tissue sections (10 *μ*m) were lysed by suspension in 20 *μ*L lysis solution [0.1 mol/L Tris‐HCl pH 8, 0.1 mol/L dithiothreitol (DTT), 4% sodium dodecyl sulphate (SDS)] and heated at 99°C for 60 min. Filter aided sample preparation (FASP) was performed as described (Wisniewski 2013). Eluted peptides were desalted and cleaned using Oasis HLB *μ*Elution plates (Waters, Milford, Mass.), and protein concentrations were measured by A280 using NanoDrop (Thermo Scientific). The samples were analyzed with a 180 min LC gradient on a Q‐Exactive HF (Thermo Scientific) connected to a Dionex Ultimate NCR‐3500RS LC system.

We used label‐free protein quantification: Raw data from the MS was processed using Progenesis LC‐MS software (version 4.0, Nonlinear Dynamics, UK) with default settings. Features were searched with searchGUI (version 2.2.2) and PeptideShaker (version 1.2.2) applying the UniprotKB/SwissProt human database (downloaded from Uniprot August 2015, 20,197 entries). Precursor mass tolerance was set at 10 parts per million, and product mass tolerance at 0.5 Dalton. Carbamidomethylation of cysteines and oxidation of methionines were set as fixed and variable modifications, respectively. Two missed cleavages were allowed, and false discovery rate was set at 1%.

### Immunohistochemistry

Immunohistochemistry (IHC) of *AXL*,* CDH1*,* MMP14,* and *VIM* was performed on 3 and 4 *μ*m thick FFPE sections following standard methodology. The following primary antibodies were used: *AXL* (1H12, BerGenBio AS, Bergen, Norway,(Nalwoga et al. 2016)), *CDH1* (monoclonal mouse anti‐E‐cadherin, clone NHC‐38, Dako, Agilent Technologies, Santa Clara, CA, USA, catalogue number: MA5‐12547), *MMP14* (monoclonal rabbit anti‐MMP14, Abcam, Cambridge, UK, catalogue number: EP1264Y) and *VIM* (monoclonal mouse anti‐vimentin, clone V9, Dako, Agilent Technologies, Santa Clara, CA, USA, catalogue number: M072529‐2). Slides were incubated with the specified antibodies, such as *AXL* 1 *μ*g/mL, *CDH1* 1:200 and *VIM* 1:1000, for 1 h at room temperature. *MMP14* antibodies 1:1200 were applied overnight at 4°C. Sections were counterstained with hematoxylin (Dako, catalogu number: CS70030‐2), then dehydrated and cover‐slipped.

## Results

The hypothesis underlying this study was that a transition from normal renal structure to ccRCC is characterized by EMT. To test this hypothesis and to characterize the extent of EMT in ccRCC, we determined gene expression levels of EMT genes from ccRCC patient biopsies and compared them to levels in matched, adjacent, normal tissue. Patient characteristics are summarized in Table 1. Importantly, no patient had signs of metastases.

### Increased generic EMT score in renal cell carcinoma

We first assessed whether gene expression changes could be used to reflect the degree of EMT in the samples. To that end, we employed an EMT score calculation, which is based on the enrichment score of 315 genes, as previously described (Tan et al. 2014). A higher EMT score indicates greater mesenchymal characteristics. Accordingly, a lower EMT score reflects a more epithelial‐like phenotype.

**Figure 1:**
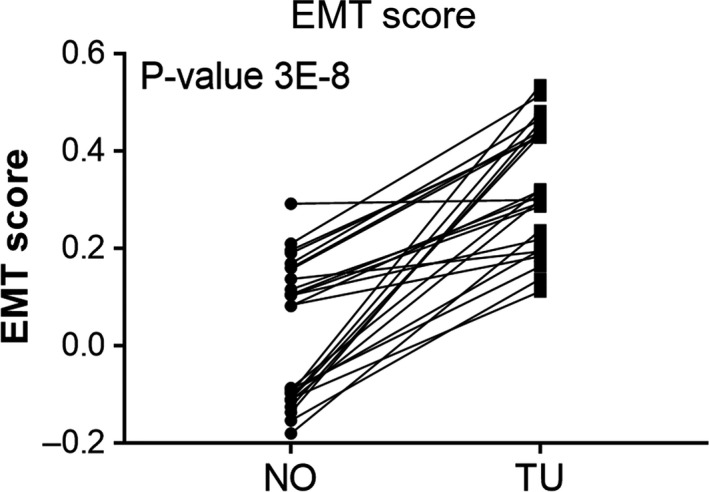
Epithelial‐Mesenchymal Transition (EMT) score of 26 adjacent normal‐tumor pairs from renal clear cell carcinoma patients. *P*‐value shown is computed by matched‐pairs two‐sided Wilcoxon signed rank test. NO, normal, non‐carcinoma tissues; TU, respective ccRCC samples.

Figure 1 shows that the EMT score of normal noncancerous kidney tissue samples indicates a relatively mesenchymal‐like value. However, in 25 of 26 sample pairs, the EMT score values were significantly higher in ccRCC (TU) samples as compared to the matched normal (NO) sample. Importantly, there was neither a difference comparing normal, non‐cancer tissues obtained from partial versus radical nephrectomy nor effects of age, sex or cancer stage on the degree of EMT score (data not shown).

The data also show that in our dataset EMT score alone cannot be used as indicator of ccRCC, since the individual scores of NO and TU samples are not clearly separated. The value of this analysis lies in the increase of the EMT score from NO to the respective matched TU sample.

### Alterations in EMT gene expression distinct renal cell carcinoma from normal tissue and correlate with cancer stage

We have investigated alterations in the gene expression pattern on the mRNA and on the miRNA level as well as to a lesser degree on the protein level.

#### Analysis of mRNA abundances

We compiled a list of EMT‐related genes from three public sources and our own data. The study by Chen et al. (2014) used microarray data to identify 46 EMT genes in normal and primary ccRCC. Gröger and colleagues performed a meta‐analysis of cancer gene expression studies in primary cell cultures and concluded on a core set of 131 EMT genes with relevance in tumor progression (Groger et al. 2012). Further we extracted 357 genes from the dbEMT database (http://dbemt.bioinfo-minzhao.org/, (Zhao et al. 2015). Surprisingly, only six genes were common to all three datasets (*CDH1*,* CDH2*,* FN*,* MMP2*,* VIM*, and *ZEB1*). The inclusion of other relevant genes, such as *GAS6* and *AKT3*, resulted in a gene list comprising 483 EMT‐related genes implicated in ccRCC. In the gene expression analysis of matched ccRCC and adjacent normal biopsy samples from 26 patients (*n* = 52 samples), 399 of these 483 EMT‐related genes (83%) passed the expression filter (see Material and Methods) and were retained in the analysis. Of those 399 genes, 137 were differentially expressed (absolute logFC>1, adjusted *P*‐ ≤ 0.05). Of the 315 genes used in the EMT score analysis, 71 were included in the list of 399 detected EMT‐related genes, and 39 were differentially expressed.

**Figure 2:**
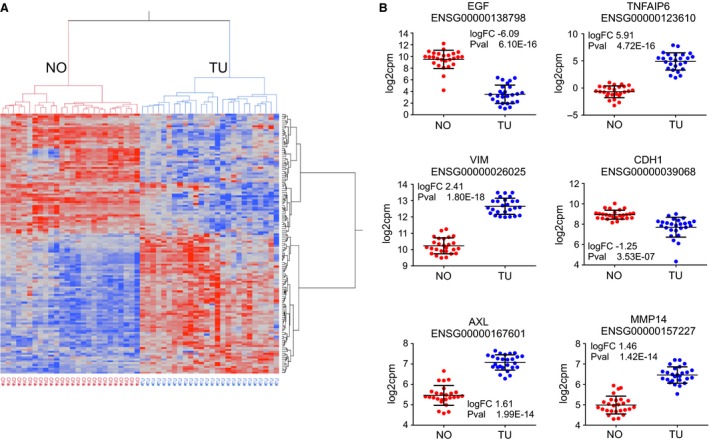
RNA sequencing data analysis. (A) Unsupervised hierarchical cluster analysis with 137 differentially expressed EMT‐related mRNA. The samples segregate into two large clusters, the normal (“NO”) samples, and the ccRCC samples (“TU”). (B) Scatterplot illustration of expression levels of selected genes.

Unsupervised hierarchical cluster analysis illustrates how the normalized expression values of the 137 mRNAs led to the formation of two distinct clusters of samples, consisting of normal and tumor samples, respectively (Fig. 2A). Genes with the largest fold changes in tumor tissue compared to normal tissue included *EGF* and *TNFAIP6*. Scatterplots for selected genes are shown in Figure 2B.

Twenty genes with the largest absolute fold change of mRNA abundance and smallest *P*‐values are shown in Table 2. From this list, three genes were detected and confirmed by proteomics, as described below. Other upregulated, important EMT‐related genes, which were not present in the list of the top 20 genes, were *SNAI1, TGFβ1, and CTNNB1 (β‐catenin);* data not shown.

**Table 2:** Differentially expressed genes

ENSEMBL_ID	Symbol	RNAseq		*t*	*P* value	adj *P* value	Proteomics		
		log FC (TU/NO)	abs ClogFC (TU/NO)						
							log FC (TU/NO)	*P* value	adj *P* value
ENSG00000138798	EGF	−6.09	6.09	—16.69	6.10E‐16	9.96E‐14			
ENSG00000123610	TNFAIP6	5.91	5.91	16.86	4.72E‐16	8.02E‐14			
ENSG00000171004	HS6ST2	−5.61	5.61	—12.87	3.53E‐13	1.06E‐11			
ENSG00000135374	ELF5	−5.54	5.54	—11.52	4.77E‐12	8.54E‐11			
ENSG00000159263	SIM2	−5.29	5.29	—10.55	3.45E‐11	4.44E‐10			
ENSG00000198910	L1CAM	−4.34	4.34	—13.61	9.37E‐14	3.85E‐12	!0.43	8.18E!03	2.77E!02
ENSG00000136943	CTSL2	−3.94	3.94	—11.14	1.02E‐11	1.59E‐10			
ENSG00000137648	TMPRSS4	−3.60	3.60	−6.34	7.96E‐07	2.90E‐06			
ENSG00000107485	GATA3	−3.59	3.59	—18.14	7.30E‐17	1.87E‐14			
ENSG00000198780	FAM169A	−3.45	3.45	—12.67	5.17E‐13	1.43E‐11			
ENSG00000146674	IGFBP3	3.38	3.38	19.75	8.31E‐18	4.18E‐15	3.26	1.23E‐03	6.70E‐03
ENSG00000113083	LOX	3.35	3.35	11.59	4.09E‐12	7.52E‐11			
ENSG00000104413	ESRP1	−2.99	2.99	−6.16	1.28E‐06	4.48E‐06			
ENSG00000106541	AGR2	−2.97	2.97	−3.76	8.03E‐04	1.60E‐03			
ENSG00000184937	WT1	−2.97	2.97	−8.16	7.88E‐09	4.72E‐08			
ENSG00000113578	FGF1	−2.95	2.95	−9.64	2.51E‐10	2.33E‐09			
ENSG00000118526	TCF21	−2.85	2.85	−9.10	8.57E‐10	6.83E‐09			
ENSG00000101144	BMP7	−2.75	2.75	−3.87	6.05E‐04	1.24E‐03			
ENSG00000112715	VEGFA	2.72	2.72	14.91	1.01E‐14	7.68E‐13			
ENSG00000038427	VCAN	2.72	2.72	7.92	1.41E‐08	7.97E‐08	3.01	3.90E‐05	6.15E‐04

Top 20 differentially expressed EMT genes (sorted by decreasing absolute log_2_fold change in mRNA abundance) and available proteomics results.

**Figure 3:**
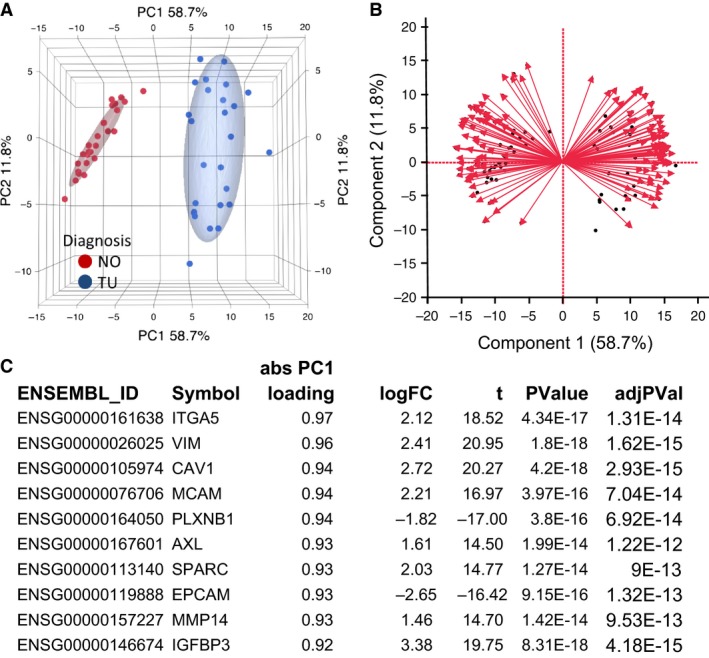
Principal component analysis with differentially expressed EMT genes. (A) Principal component analysis (PCA) with 137 differentially expressed EMT‐mRNA. The sample groups “healthy” (normal, NO) and “ccRCC” (tumor, TU) are separated along the principal component 1 (PC1). Ellipsoids indicate the 95th percentile of data points per group. (B) The biplot demonstrates the samples as dots, and the contribution of each variable toward the explanation of the variance of the data as red arrows. Each arrow can be attributed a value, the loading score. (C) Ten mRNA with the largest absolute loading scores. ITGA5, AXL and MMP14 are among the mRNAs with the largest loading scores.

To analyze the contribution of the 137 variables (differentially expressed genes) toward explaining the data structure (i.e., variance), we applied principal component analysis (PCA), as depicted in Figure 3. The normalized expression data for the differentially expressed mRNA explained 58.7% of the variance in principal component 1 (PC1) and leads to a visual separation of the normal and the tumor samples (Fig. 3A). The biplot in Figure 3B represents both the samples (illustrated as black points) and vectors of the variable contribution (or loadings, in red) to represent the data structure. As in the PCA, the axes of the biplot are the principal components. Vectors that point in the same direction correspond to variables that have similar response profiles. The loadings represent correlations between the input variables and the principal component scores; they can be negative or positive. Extraction of the loadings of the variables for PC1, and ranking them by the largest to smallest absolute value identifies the mRNA variables with the largest contribution to PC1, which explains the difference between normal and tumor samples. As shown in the list of ten mRNA with largest absolute loadings for PC1 (Fig. 3C), *ITGA5* has the largest contribution, *AXL* and *MMP14* rank 6th and 9th, respectively, indicating their importance in explaining the data structure, similarity and difference between samples.

**Figure 4:**
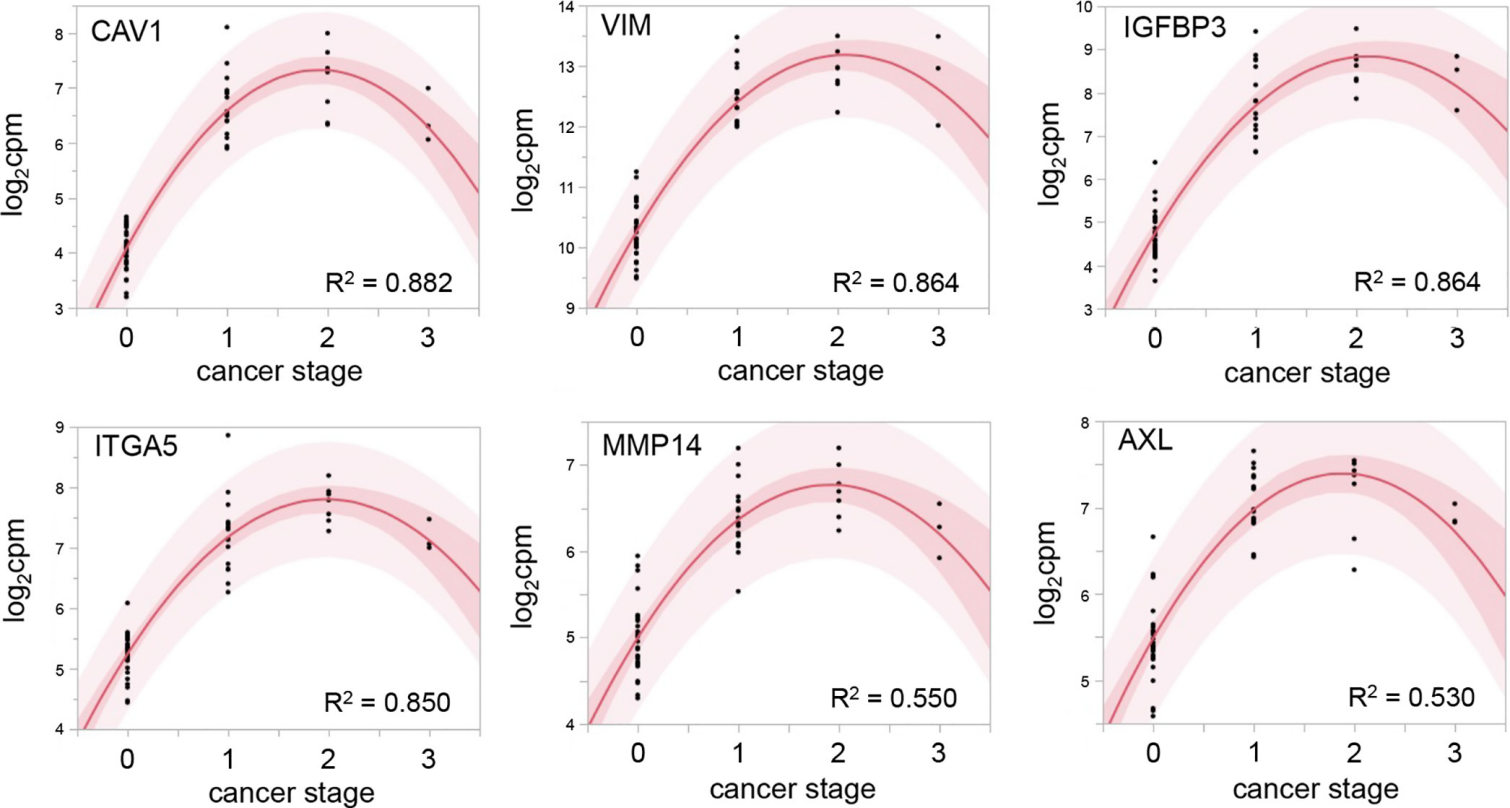
Bivariate polynomial regression of the mRNA abundance and cancer stage. *CAV1*,*VIM*,*IGFBP3*, and *ITGA5* were the mRNAs with strongest regression. The expression level for all mRNAs declined in samples with the highest tumor stage. The dark red area is the confidence limit for the expected fitted mean, the light red area displays the confidence limits for the individual predicted value. The confidence limits reflect variation in the error and variation in the parameter estimates. Cancer stage (see also Table 1) is indicated as numerical values from 1 to 3, with 0 indicating tumor‐unaffected status corresponding to the respective noncancerous normal tissues.

Next, we analyzed whether any mRNA expression profiles were correlated with cancer stage, as described by us and others (von Roemeling et al. 2014; Eikrem et al. 2016a). We correlated the log2 cpm expression values of the EMT genes, which were obtained from the LIMMA/VOOM analysis in R Bioconductor, to the cancer stage applying a bivariate polynomial fitting algorithm. *CAV1* had the largest absolute correlation, followed by *VIM*,* IGFBP3*, and *ITGA1*. Interestingly, the expression levels did not increase from the stage 2 to stage 3, and in some cases even decreased. These results are depicted in Figure 4.

**Figure 5:**
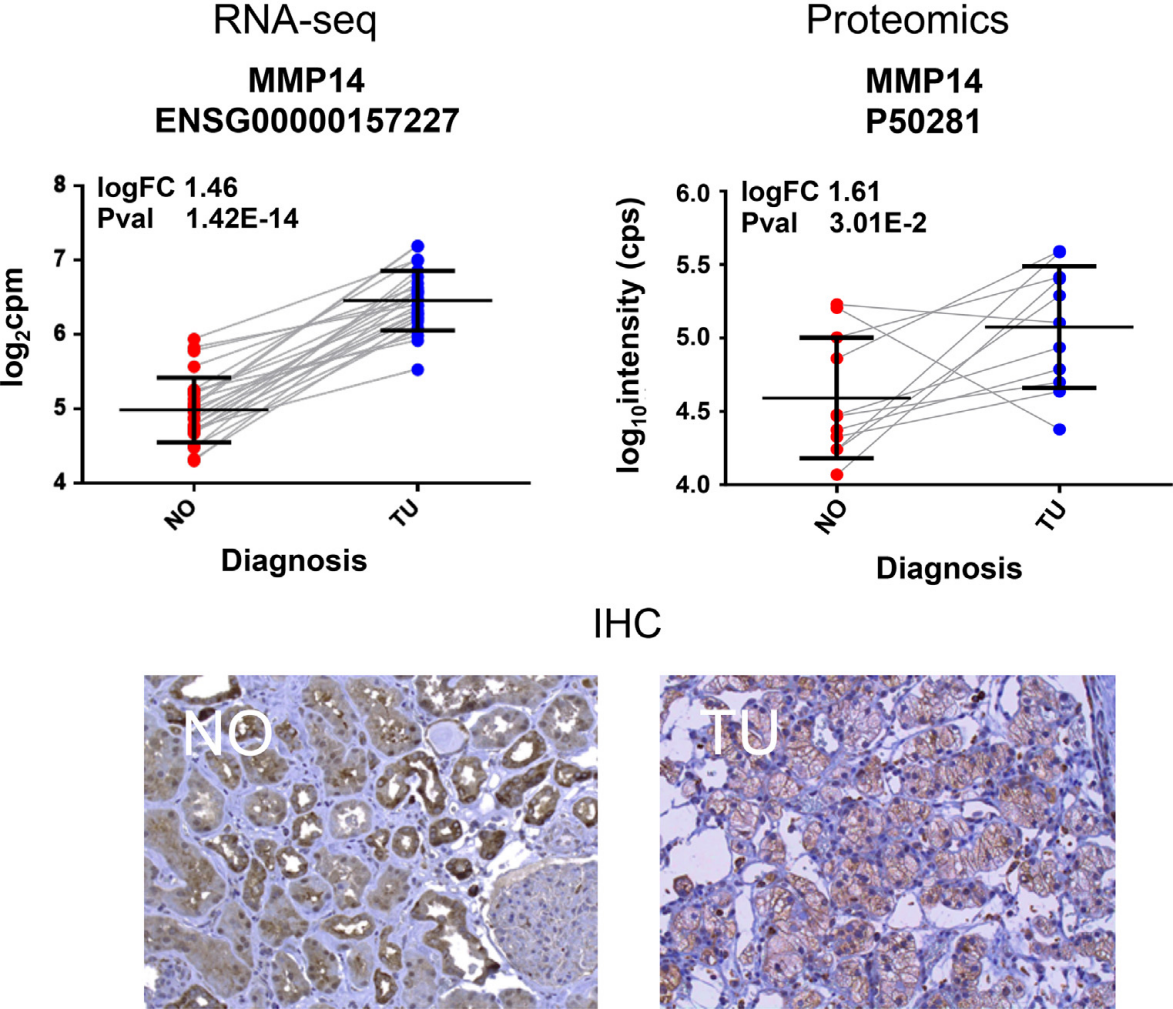
Detection of expression changes of MMP14 in ccRCC. Top left (“RNA‐seq”): MMP14 mRNA is about 1.46‐logfold increased in ccRCC. Matched normal samples from the same patients are connected by a line to illustrate the expression change. Top right (“Proteomics”): Increased abundance of MMP14 protein in ccRCC. Two samples, patients 27 and 29, showed decrease in MMP14 expression in ccRCC. In RNA sequencing data, *MMP14* mRNA levels were increased in ccRCC also for these two patients. Matched normal and ccRCC samples from the same patient are connected to illustrate the expression change. Bottom (“IHC”): Representative immunohistochemistry (IHC) results showing the increased detection of MMP14 epitope in an ccRCC sample (“TU”) in comparison to the matched healthy (“NO”) sample from the same patient.

Figure 5 summarizes the expression data obtained for *MMP14*. Besides RNA‐sequencing, we applied proteomics, and immunohistochemistry. Using these three technologies, we could confirm RNA abundance as well as protein abundance of MMP14, revealing an approximate twofold higher average expression level in ccRCC tumor specimens compared to normal samples. Note, only one patient showed an inexplicable decrease on MMP14 in ccRCC as analyzed by proteomics.

**Figure 6:**
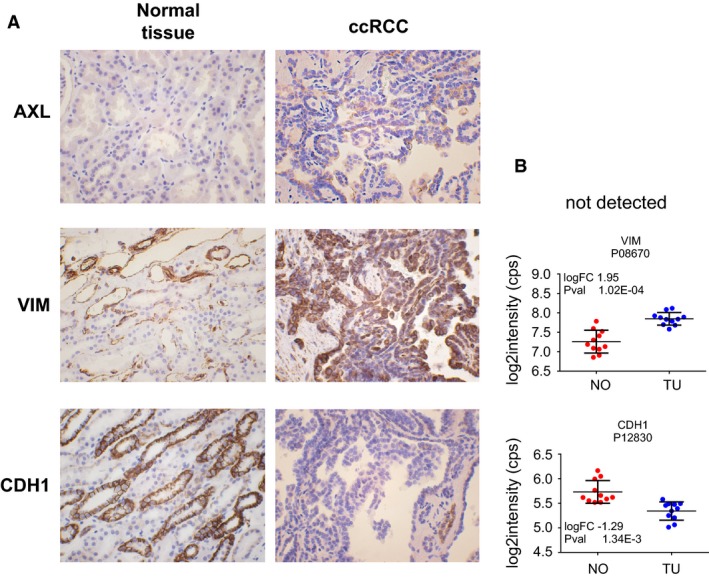
Representative immunohistochemistry (IHC) analyses of protein abundance by proteomics of three genes involved in EMT. Protein level of AXL and VIM were increased in ccRCC, while CDH1 protein was decreased (A). IHC results are supported by proteomics data for VIM and CDH1 (B). AXL protein was not detected in the proteomics dataset.

Applying proteomics and IHC, we also detected increased abundance of protein of AXL, and VIM, in ccRCC, and decreased abundance of CDH1, thereby supporting RNA sequencing data, as depicted in Figure 6A and B. In addition, Figure 6A indicates that there could be some stromal cells detectable next to *CDH1*‐positive tubules, which may explain in part the relatively high EMT values of some carcinoma‐adjacent normal kidney samples (Fig. 1).

#### Analysis of microRNA expression

**Figure 7:**
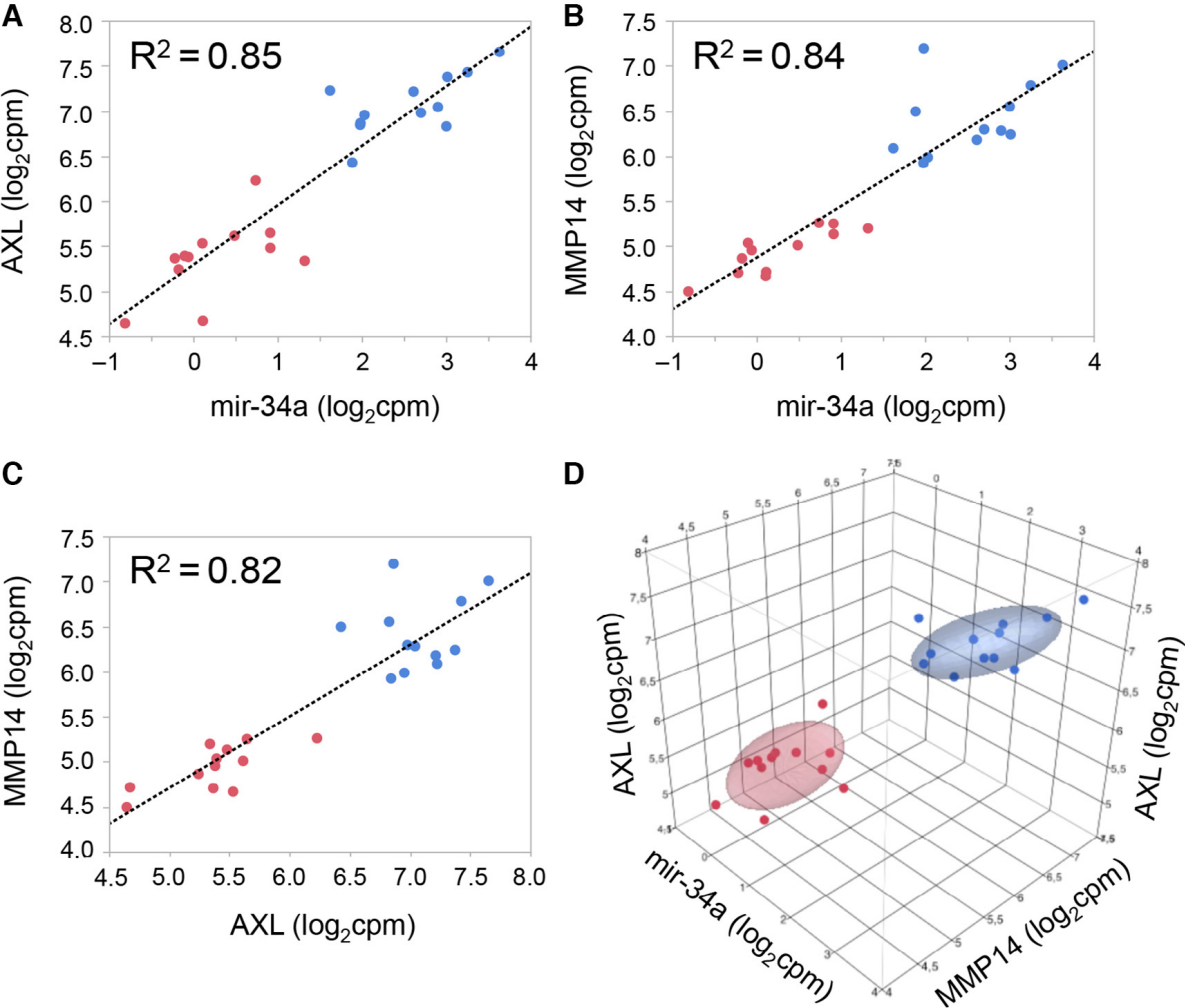
Correlation of expression levels of miR‐34a and target genes *AXL* and MMP14. Pearson correlation of expression levels of miR‐34a and the target genes *AXL* (A) and *MMP14* (B), of *AXL* and *MMP14* (C), and all mir‐34a and *AXL* and *MMP14* (D). Red dots indicate normal samples, blue dots ccRCC tumor samples. Ellipsoids indicate the 95th percentile of data points per group.

Expanding our analysis of gene expression data to the analysis of those microRNAs with involvement in EMT, we sequenced microRNAs from 12 ccRCC samples and the 12 respective normal specimens from our patient cohort, followed by pairwise comparison of their abundance as done for mRNA. Most of the patient material is shared between the microRNA and the mRNA studies. MicroRNAs associated with EMT were identified from the literature (Zhang and Ma 2012; Zaravinos 2015). The respective results are shown in Table 3. Mir‐34a, which targets AXL and MMP14 (Jia et al. 2014; Li et al. 2015) is one of the most significantly affected microRNAs. The expression levels of these three microRNA and mRNA species are found to be strongly positively correlated in our datasets (Fig. 7).

**Table 3:** Differentially expressed microRNA (mir)

microRNA	micro RNA				ENSEMBL ID	Target mRNA			
	Precursor	FC (TU vs. NO)	*P* value	adj *P* value		Symbol	FC (TU/NO)	*P* value	adj *P* value
hsa‐miR‐200b‐3p	hsa‐mir‐200b	−2.53	2.99E‐04	8.95E‐04					
hsa‐miR‐200b‐5p	hsa‐mir‐200b	−2.34	1.48E‐04	5.10E‐04	ENSG00000039068	CDH1	−2.38	3.53E‐07	1.40E‐06
hsa‐miR‐200c‐3p	hsa‐mir‐200c	−39.12	6.84E‐09	3.59E‐07	ENSG00000169554	ZEB2	1.54	4.38E‐07	1.71E‐06
hsa‐miR‐141‐5p	hsa‐mir‐141	−30.31	1.25E‐07	2.29E‐06	ENSG00000148516	ZEB1			
hsa‐miR‐141‐3p	hsa‐mir‐141	−17.64	1.08E‐07	2.18E‐06	ENSG00000168036	CTNNB1	1.16	3.20E‐03	5.71E‐03
hsa‐miR‐429	hsa‐mir‐429	−2.34	4.36E‐05	1.83E‐04					
hsa‐miR‐30a‐3p	hsa‐mir‐30a	−3.09	4.54E‐07	5.87E‐06					
hsa‐miR‐30a‐5p	hsa‐mir‐30a	−2.32	1.50E‐05	7.48E‐05					
hsa‐miR‐34a‐3p	hsa‐mir‐34a	4.97	7.79E‐08	2.04E‐06	ENSG00000167601	AXL	3.05	1.99E‐14	1,22E‐12
hsa‐miR‐34a‐5p	hsa‐mir‐34a	2.73	4.66E‐06	2.80E‐05	ENSG00000157227	MMP14	2.74	1.42E‐14	9.53E‐13
hsa‐miR‐34c‐5p	hsa‐mir‐34c	2.55	1.42E‐02	2.58E‐02					
hsa‐miR‐203a‐3p	hsa‐mir‐203a	−2.26	1.44E‐02	2.60E‐02	ENSG00000124216	SNAIL1	1.78	1.21E‐03	2.33E‐03
hsa‐miR‐10b‐5p	hsa‐mir‐10b	−2.72	1.44E‐09	1.26E‐07	ENSG00000128710	HOXD10	−2.82	1.06E‐08	6.13E‐08
					ENSG00000156299	TIAM1	1.92	5.66E‐09	3.55E‐08
hsa‐miR‐21‐3p	hsa‐mir‐21	6.23	1.05E‐07	2.18E‐06	ENSG00000150593	PDCD4	1.35	5.55E‐07	2.11E‐06
					ENSG00000140416	TPM1	1.79	6.67E‐07	2.47E‐06
hsa‐miR‐31‐3p	hsa‐mir‐31	−3.07	8.92E‐03	1.71E‐02	ENSG00000156299	TIAM1	1.92	5.66E‐09	3.55E‐08
hsa‐miR‐155‐5p	hsa‐mir‐155	9.62	5.31E‐08	1.86E‐06	ENSG00000067560	RHOA	1.16	3.05E‐04	6.59E‐04
hsa‐miR‐194‐3p	hsa‐mir‐194‐2	−3.21	3.47E‐04	1.00E‐03	ENSG00000170558	CDH2	1.79	2.69E‐05	7.13E‐05
					ENSG00000134873	CLDN10	−4.95	8.69E‐11	9.58E‐10
hsa‐miR‐204‐5p	hsa‐mir‐204	−4.25	1.50E‐04	5.14E‐04	ENSG00000113946	CLDN16	−25.37	5.29E‐10	4.48E‐09
hsa‐miR‐204‐3p	hsa‐mir‐204	−3.96	4.97E‐05	2.02E‐04	ENSG00000164007	CLDN19	−26.79	8.10E‐13	2.00E‐11
					ENSG00000105329	TGFB1	2.80	6.14E‐14	2.77E‐12
					ENSG00000124216	SNAIL1	1.78	1.21E‐03	2.33E‐03
hsa‐miR‐138‐5p	hsa‐mir‐138‐2	−12.09	2.01E‐05	9.40E‐05	ENSG00000039068	CDH1	−2.38	3.53E‐07	1.40E‐06
hsa‐miR‐138‐5p	hsa‐mir‐138‐1	−12.01	1.78E‐05	8.72E‐05					
hsa‐miR‐9‐5p	hsa‐mir‐9‐1	−6.32	2.73E‐04	8.36E‐04					
hsa‐miR‐10a‐5p	hsa‐mir‐10a	−3.77	1.12E‐06	1.05E‐05					
hsa‐miR‐10a‐3p	hsa‐mir‐10a	−2.71	1.86E‐05	8.94E‐05					
hsa‐miR‐335‐5p	hsa‐mir‐335	−2.85	2.03E‐05	9.40E‐05					

Relevant microRNA in EMT. Expression changes of microRNAs and their target mRNAs in ccRCC compared with normal tissue.

#### Expression of tumor EMT‐genes and also of fibrosis‐related genes correlates with patient survival

**Figure 8:**
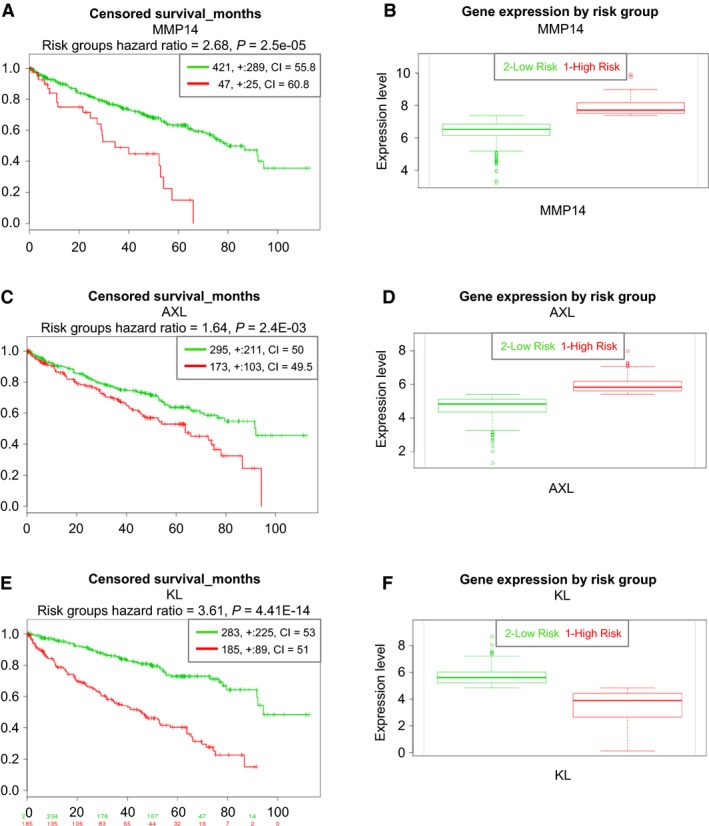
Survival analysis for patients with ccRCC based on individual genes. Higher mRNA expression levels of *MMP14* (A and B), and *AXL* (C and D) are significantly associated with worse survival. Higher *KL* mRNA expression levels are significantly associated with improved patient outcome (E and F).

However, while mir‐34a is highly expressed in high‐risk ccRCC groups, it did not correlate with patient survival in 217 ccRCC samples (see Material and Methods; data not shown). *MMP14* and *AXL* on the other hand are significantly associated with patient survival, being higher expressed in high‐risk groups within 468 human ccRCC samples (Fig. 8A–D).

*KL* (klotho) has recently been shown to inhibit TGF*β*1 and to decrease renal fibrosis and cancer metastasis (Doi et al. 2011). As *KL* was differentially expressed in our dataset (fold change TU/NO ‐3.43, *P*‐value 2.15E‐07), we tested the effect on patient survival on 468 samples of the TCGA clear cell carcinoma dataset implemented (Material and Methods). In accordance with the literature, lower expression levels of *KL* indicated lower patient survival (Risk Group Hazard Ratio 3.61, *P*‐value 4.41E‐14), as shown in Figure 8E–F.

**Figure 9:**
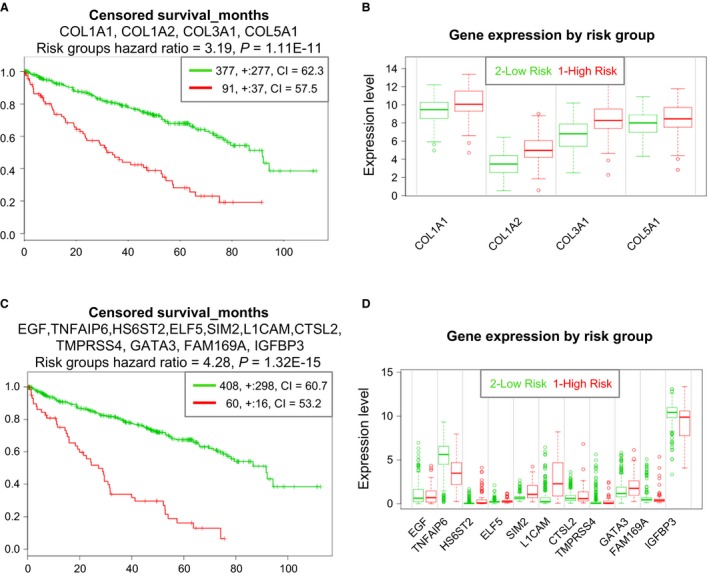
Survival analysis for patients with ccRCC based on two gene panels. Performance of a panel of four collagen genes with co‐expression in the matched pairs data (A and B). Performance of a panel of eleven genes with superior rank hazard ratio, and their expression level in risk groups (C and D). The genes have been selected based on their performance in the survival analysis from a set of 20 genes with highest TU/NO‐fold change listed in Table 2.

Finally, we evaluated combinations of differentially expressed genes. A set of four collagen genes with an expression very tightly correlated across the samples (*COL1A1*,* COL1A2*,* COL3A1*, and *COL5A1*; correlation 0.8 ± 0.025) outperformed *MMP14* and *AXL* (Risk Group Hazard Ratio 3.19, *P*‐value 1.11E‐11). All collagen genes are higher expressed in high‐risk group and correlated with patient survival (Figure 9A and B).

Furthermore, we tested various combinations of genes from the set of 20 genes highest fold changes between NO and TU (Table 2). A set of 11 genes allowed us to predict patient survival to a much better degree than any individual gene we tested (Risk Group Hazard Ratio 4.28, *P*‐value 1.32E‐15), as shown in Figure 9C and D.

The member genes of two gene sets, the collagen‐based set (Fig. 9A and B) and the panel of 11 genes (Fig. 9C and D), underscore the tight link between EMT and fibrosis development. Notably, EMT and its intermediate states have recently been identified as key promoters of organ fibrosis (Nieto et al. 2016). Amongst the genes of the two sets, especially collagens 1 and 3, but also 5 can be augmented in solid organ fibrosis, including the kidney (Mak et al. 2016). Moreover, the EMT drivers TGF*β* and epithelial growth factor (EGF) are also linked to the promotion of fibrosis development (Kok et al. 2014; Grande et al. 2015).

**Figure 10:**
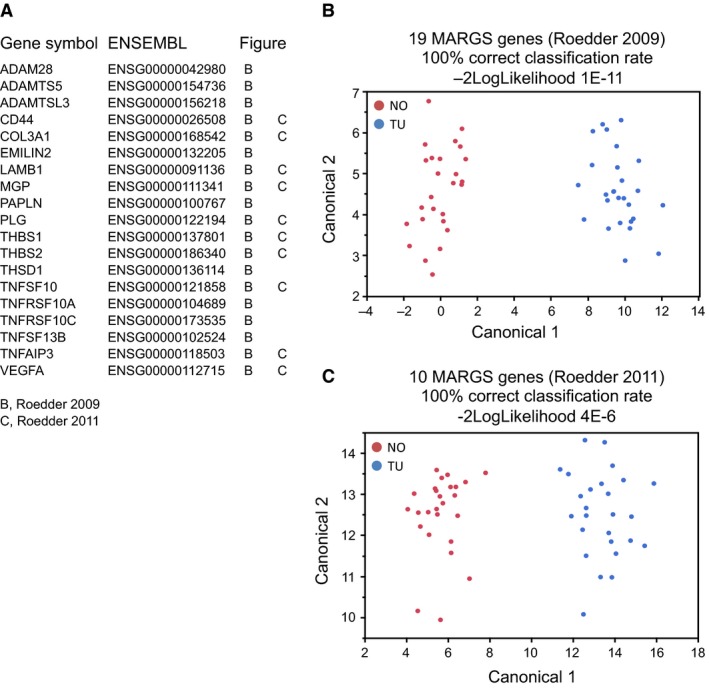
Fibrosis classifier. Two MARGS‐based classifiers of fibrosis are diagnostic of ccRCC. Most of the genes of the classifier panels which had been developed to diagnose interstitial fibrosis in renal allografts (19 genes, (Rodder et al. 2009)), and solid organ fibrosis (10 genes, (Rodder et al. 2011)) are differentially expressed in the ccRCC dataset (A). Applying linear discriminant analysis with the 19‐gene panel (B) or with the 10‐gene panel (C) has a 100% correct classification rate, and indicates a role of fibrosis in ccRCC.

Therefore, in a subsequent step, we applied our previously described fibrosis classifier in humans, which is based on metzincins and related genes (MARGS). The classifier was successfully applied in renal allografts (classifier with 19 MARGS genes) and in solid human organ fibrosis (classifier with 10 of the 19 MARGS genes), (Rodder et al. 2009, 2011). This fibrosis classifier was also validated across species in rat fibrosis models (Marti et al. 2014, 2016). Both models classified all samples correctly into normal and ccRCC samples (Fig. 10) despite the fact that the majority of our tumor samples (*n* = 24) had areas of intra‐tumor stroma and/or evidence of fibrosis, as judged by light microscopy (data not shown).

We also tested the performance of the MARGS classifiers with respect to the patient survival. The 19‐gene panel classifier had a Risk Group Hazard Ratio of 3.6 (*P*‐value 4E‐12), and the 10‐gene MARGS classifier of solid organ fibrosis a Risk Group Hazard Ratio of 2.97 (*P* ‐value 7.3E‐9).

## Discussion

This study provides additional evidence for the pathophysiologic and prognostic role of EMT gene expression in the development of ccRCC. To the best of our knowledge, this is the first report combining mRNA/miRNA next generation sequencing with proteomics in FFPE ccRCC tissues, and linking results to fibrosis. Firstly, we observed an increased EMT score in ccRCC. Thereafter, we have analyzed several individual genes and finally, we have obtained two prognostic EMT‐related gene sets consisting of four and eleven genes, respectively. Many of these genes are also linked to fibrosis development.

Numerous genes are involved in EMT and their selection for analysis ultimately remains incomplete. We decided to first use an established, generic EMT score for an initial EMT screening, as published by members of our group (Tan et al. 2014). The respective results indicated that our dataset can be used to address whether EMT‐related genes play a role in ccRCC. It also demonstrates that samples adjacent to tumor and histologically cancer‐negative can display a gene expression pattern consistent certain degree of EMT development.

Thereafter, detailed gene expression analyses were primarily based on the dbEMT database containing 357 genes, as this is a curated gene list based on experimental evidence extracted manually from respective publications.

EMT indeed plays an important role in renal cell carcinoma, which supports the ultimate goal and rationale for EMT‐directed therapeutic strategies for these patients (Piva et al. 2016). Our comprehensive approach is best exemplified by the description of the up‐regulated MMP14 by investigations on all levels, such as mRNA sequencing, proteomics, and immunohistochemistry, as well as by the analysis of its regulator miR‐34a. Notably, MMP14 is ‐ together with AXL, caveolin 1 (CAV1) and ITGA5 ‐ among the top genes contributing to the differentiation of cancer from normal tissue. Expression levels of miR‐34a and target genes AXL and MMP14 are closely correlated among each other and clearly separate tumor from adjacent normal tissue.

The integrin ITGA5 is expressed by renal carcinoma cells but its exact function remains to be determined (Poplawski et al. 2017). Based on our findings, this molecule warrants further exploration. CAV1 expression displayed the largest absolute correlation with tumor stage, followed by VIM, IGFBP3, and ITGA1. CAV1 has not been described in the context of EMT in ccRCC, but it is over‐expressed in hepatocellular carcinoma and promotes cancer cell invasion via inducing EMT (Gai et al. 2014). Accordingly, increased expression of CAV1 can predict a poor prognosis of patients with ccRCC (Steffens et al. 2011).

Our results have implications for prognosis of ccRCC. Higher *MMP14* and *AXL* mRNA levels are associated with lower patient survival. Nevertheless, their common regulator mir‐34a, although highly produced in high‐risk ccRCC, does not have prognostic value in terms of patient survival. In accordance with our results, AXL inhibition represents an emerging treatment option in oncology (Feneyrolles et al. 2014). A selective small molecule Axl kinase inhibitor (BGB324) is currently in Phase II clinical trials for acute myeloid leukemia, melanoma and non‐small cell lung cancer. AXL inhibition in experimental ccRCC models is beneficial indicating a new therapeutic option (Yu et al. 2015).

AXL and miR‐34a are engaged in a complex auto‐regulation circuit, which involves targeting of AXL by miR‐34a, while at the same time AXL overexpression leads to increased expression of miR‐34a via ELK1 (Cho et al. 2016). These data may help explain the concordant up‐regulation of AXL and miR‐34a in our datasets.

Contrary to our data, miR‐34 expression levels did not correlate to AXL mRNA abundance in a previous ccRCC investigation (Fritz et al. 2015). We do not have a clear explanation for this discrepancy beyond the known ccRCC heterogeneity. However, in that study, levels of miR‐34a were also increased in ccRCC but not associated with patient outcome, as it is the case for the present investigation (Fritz et al. 2015).

MMP14 represents an increasingly recognized but complex mediator of EMT and ccRCC. MMP14 is a known target of miR‐34a (Jia et al. 2014). In our analysis, we observe positive correlation between the expression levels of *MMP14* and miR‐34a. This could be explained by the fact that MMP14 is not exclusively regulated by miR‐34a. In this respect, we must consider the complex network of miR‐34a, which targets several MMPs, including MMP14, and represses their expression. Higher expression of another microRNA, miR‐21, decreases the expression of genes coding for TIMPs, such as TIMP3, which then in turn leads to an increase in invasion‐promoting MMPs (Chernov and Strongin 2011). Importantly, TIMP3 is a strong inhibitor of MMP14 (Will et al. 1996). Thus, the MMP regulation pattern is not always linear and unidirectional, but rather includes several checkpoints and multi‐directional paths. Furthermore, control of MMP expression and activity is remarkably complex, including at the level of transcription, cellular compartmentalization, zymogen activation, and enzyme inhibition (Baker et al. 2002). MMP14 is also connected to EGFR, since MMP14 exerts a positive effect on the MEK1/MAPK axis through transactivation of the EGFR (Mahimkar et al. 2011). Indeed, *EGFR* is more than twofold up‐regulated in our ccRCC patient cohort (data not shown). The expression level of *EGFR* was previously found to be associated with high ccRCC tumor grade and worse prognosis (Dordevic et al. 2012; Minner et al. 2012).

Klotho (KL) can affect EMT via an interaction with SNAIL. *KL* inhibits the PI3K/Akt/GSK3beta/Snail pathway and decreased *KL* expression negatively correlates with ccRCC patient survival in the literature (Zhu et al. 2013; Gigante et al. 2015). In this respect, Klotho reduces epithelial‐mesenchymal transition and cellular invasion in renal cell carcinoma (Zhu et al. 2013). Thus, our data regarding higher *KL* expression favoring better patient survival are in accordance with the literature. *KL* is a gene encoding a transmembrane protein with anti‐aging and tumor suppression functions (Kuro‐o et al. 1997; Xie et al. 2013; O'Sullivan et al. 2016). In kidneys, *KL* is naturally present in the proximal tubules, where it increases phosphaturic processes by modulating the activity of renal phosphate transporters. Decreased expression levels of *KL* are also associated with oxidative stress (O'Sullivan et al. 2016).

Despite observations that many individual genes can be used at the mRNA level to indicate better or worse prognosis, a combined set comprising several genes may eventually give the best predictive value. Accordingly, we have designed the two novel candidate prognostic marker sets representing mRNA abundances of a limited number of genes to best predict patient survival.

Our results further support a close relationship between EMT and fibrosis development. Several genes of our prognostic gene sets, such as collagens and EGF, are known to bridge EMT and fibrosis. Accordingly, our previously described MARGS‐based fibrosis classifier identified all our cancer samples correctly and was also correlated with patient survival. Our results may lead to better early identification of tissues undergoing EMT and potentially fibrosis in the onset of ccRCC, and they support development of new treatment avenues. Nevertheless, our study has some limitations. The sample size of kidney biopsies (*n* = 52) from our ccRCC patient cohort is limited. Furthermore, there is cellular heterogeneity in the tumors and adjacent normal tissues. Hence, our results are a summation of this variability, but reflecting the real life clinical situation. The issue of cell‐specificity regarding gene expression must be addressed in the future by tissue microdissection or even by single cell sequencing.

## Conclusion

EMT linked to fibrosis represents a prominent feature in ccRCC, which is closely associated with worse patient survival. Therefore, EMT‐related genes represent a promising target for future therapeutic interventions.

## Conflict of Interest

No disclosures and no conflict of interest to be reported.
