# Corrigendum

**DOI:** 10.14814/phy2.13671

**Published:** 2018-04-24

**Clear Cell Renal Cell Carcinoma is linked to Epithelial‐to‐Mesenchymal Transition and to Fibrosis**

Lea Landolt, Øystein Eikrem, Philipp Strauss, Andreas Scherer, David H. Lovett,

Christian Beisland, Kenneth Finne, Tarig Osman, Mohammad M. Ibrahim, Gro Gausdal,

Lavina Ahmed, James B. Lorens, Jean Paul Thiery, Tuan Zea Tan, Miroslav Sekulic &

Hans‐Peter Marti

Physiol Rep, 5 (11), 2017, e13305, https://doi.org/10.14814/phy2.13305

Figure 6 supplied in the article was incorrect. The correct version is given below.

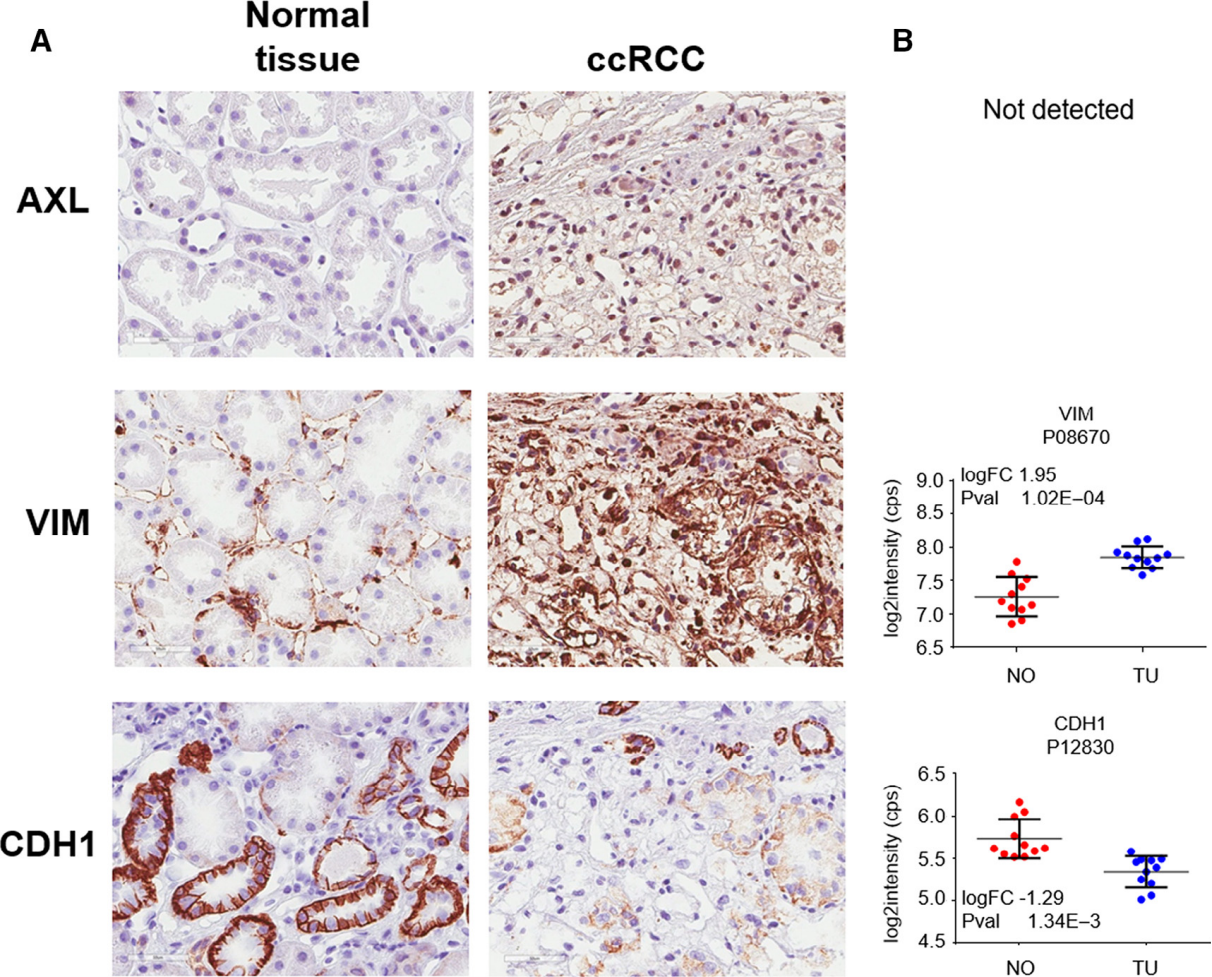

The authors apologise for the error.
